# The Effect of Epigallocatechin Gallate on the Dentin Bond Durability of Two Self-etch Adhesives

**Published:** 2015-06

**Authors:** Zahra Khamverdi, Loghman Rezaei-Soufi, Tayebeh Rostamzadeh

**Affiliations:** 1 Dental Research Center, Dept. of Operative Dentistry, School of Dentistry, Hamedan University of Medical Sciences, Hamedan, Iran;; 2 Dept. of Operative Dentistry, School of Dentistry, Gilan University of Medical Sciences,Gilan, Iran;

**Keywords:** Epigallocatechin Gallate, Dentin, Matrix Metalloproteinase, Adhesives

## Abstract

**Statement of the Problem:**

Self-etch adhesives can activate matrix metalloproteinase (MMP) which hydrolyzes organic matrix of demineralized dentin. Epigallocatechin gallate (EGCG), especially found in green tea, could inhibit the activation of MMP.

**Purpose:**

The aim of this study was to evaluate the effect of adding Epigallocatechin gallate (EGCG) into two types of adhesives on dentin bond strength.

**Materials and Method:**

In this experimental study, 64 extracted third molars were randomly divided into 16 groups. Clearfil SE Bond and Filtek Silorane System with 0 µM, 25µM, 50µM, and 100µM concentration of 95% EGCG were used for bonding. Following the bonding and fabrication of beams (1±0.1 mm^2^) and storage in distilled water, the specimens were subjected to thermal cycles. Microtensile bond strengths of 8 groups were examined after 24 hours and others were tested after 6 months. The fracture modes of specimens were evaluated by stereomicroscope and SEM. Data were analyzed by three-way ANOVA and t-test *(α = 0.05).*

**Results:**

The results of the three- way ANOVA test showed that types of bonding, storage time and interactive effect of EGCG concentration and bonding influenced the bond strength of specimens significantly (*p<*0.05). The results of the t-test indicated that storage time only had significant effect on bond strength of Clearfil SE Bond with no EGCG (*p*= 0.017). The most common failure modes in Filtek Silorane System groups and Clearfil SE Bond groups were adhesive and mixed/cohesive, respectively. The results of SEM at different magnifications showed that most fractures have occurred in the hybrid layer.

**Conclusion:**

Although adding 100 µM volume of EGCG to Clearfil SE Bond can preserve the dentin bond, incorporation of EGCG in the silorane system, especially in high concentrations, decreases the bond strength after 6 months.

## Introduction

In recent years, development of dentin bonding agents has been considerably accelerated. A widely noted important aspect is to increase the durability of resin adhesives to dentin. Bond degradation occurs through water sorption,[1] hydrolysis of ester linkages of methacrylate resins,[2] or activation of endogenous dentin matrix metalloproteinases (MMPs).[3] MMPs are a group of host-derived proteolytic enzymes which are trapped within mineralized dentin matrix and are able to hydrolyze the organic matrix of demineralized dentin. Odontoblasts produce a large number of MMPs in form of proenzymes. After activation, these proenzymes degrade extracellular matrix components. Unfortunately, self-etch and etch-and-rinse adhesives can activate MMPs.[4] Clinically applied self-etching dental adhesives are acidic (pH= 1.5–2.7) and can activate the MMPs in dentin without denaturing these enzymes during the bonding procedure that results in a 14- to 15-fold increase in collagenolytic activities.[5] Moreover, it has been proved that zoledronate, a third generation bisphosphonate, has the ability to inhibit MMP proteolytic activities.[6-7] Soybean unsaponifiables, avocados,[8] and oleic acid[9] have demonstrated effective MMP- inhibition *in vitro*. These polyphenols are used in dentistry to prevent loss of alveolar bone in periodontal diseases, to reduce dentin loss in erosion and abrasion, and to prevent caries. The mechanism has been suggested to be due to the inhibitory effect of polyphenol metalloproteinases.[10] Green tea polyphenols, especially epigallocatechin gallate (EGCG), are able to inhibit the activation of proMMP-2, MMP-2 and MMP-9.[11-12] There are not enough data in the literature about the effect of EGCG as an MMP inhibitor on durability of the bond between resin adhesives and dentin. Therefore, the incorporation of EGCG into the adhesives may positively affect dentin bond strength. Thus, the short-term effect of the preservation of dentin bond strength based on adding green tea polyphenol epigallocatechin gallate should be tested. Green tea polyphenol epigallocatechin gallate (EGCG) was added to two types of two-step self-etching adhesives in order to investigate the preservation of dentin micro tensile bond strength (μTBS) in short-term.

## Materials and Method

Two types of two-step self-etching primer adhesives, Clearfil SE Bond (Kuraray; Osaka, Japan) and Filtek Silorane System (Filtek; 3M/EPSE, USA) were used in this *in vitro *experimental study. Extract of EGCG 95% (SIGMA_ALDRICH Co.; USA) was used to prepare different concentration of EGCG including 0, 25, 50, and 100 µM through dilution in water-ethanol solution (1:1). Prepared solutions were added to the primer of Clearfil SE Bond (Kuraray; Osaka, Japan) and Silorane System adhesive (Filtek; 3M/EPSE, USA) adhesives. Then, they were mixed by Universal Ultrasonic Cleaner (DSA 100-SK2; Fujian Yu De Trade Co., China) for repeated intervals of 30 seconds of mixing and 30 seconds of rest time for 4 minutes.[13] In order to measure the degree of conversion, small amounts of adhesives were put on a polyethylene film and their solvents were gently evaporated for 10 seconds. A low-pressure air stream was applied before the second film was placed on it to form a very thin layer. The sandwich was placed into the FTIR spectrometer’s sample holder and the absorbance peaks were obtained. Then the samples were light-cured for 40 seconds by means of a dental light source with an irradiance intensity of 450mW/cm2 (Hilux LED 550; Benlioglu Dental, Ankara, Turkey), and the peaks were measured for the cured samples. All mentioned absorbance peaks were obtained through transmission mode of FTIR. The degree of conversion (DC %) was determined by the ratio of absorbance intensities of aliphatic C=C (peak at 1638cm−1) before and after the specimens were cured. The internal reference employed was aromatic C. . .C (peak at 1608cm−1). The calculation formula of the degree of conversion was as presented below:[14]

DC % = (1 − (1638cm_1/1608cm−1) peak area after curing/ (1638cm−1/1608cm−1) peak area before curing) × 10 Sixty four unerupted caries-free third molars were collected after an informed consent was obtained from the patients. These molars were stored in 0.2% thymol solution at 37ºC for less than one month. The occlusal enamel surfaces of the teeth were removed until the diameter of the exposed dentin surfaces was 5 mm.

In order to form a homogenous smear layer on the surface of the occlusal dentin, the teeth were polished with a wet 600-grit silicon carbide paper for 30 seconds. 

Filtek Z250 (3M/ EPSE, USA) composite resin was used for build-up in the four groups that were bonded with Clearfil SE Bond system. Filtek P90 (3M/ EPSE, USA), a low shrinkage composite resin was used for the remaining groups. Five layers of composite were added to the bonded surfaces and each layer was individually light-cured for 20 seconds for Filtek Z250 and 40 seconds for Filtek P90 using an LED light-curing unit (Hilux LED 550; Benlioglu Dental, Ankara, Turkey) with an output of 450 mW/cm[2]. Then, the teeth were stored in distilled water at 37ºC until the examination time. To produce a series of 1±0.1 mm^2^ beams, the teeth were longitudinally sectioned using a cutting machine (Isomet 1000; Buehler Ltd., Lake Bluff, IL, USA).

Each tooth was devided into three beams and stored in distilled water at 37ºC. Half of the specimens were tested after 24 hours (control group), while the other half (experimental group) were subjected to 2500 thermal cycles (5ºC/55ºC; dwell time 30 seconds) and were tested after 6 months. During this period, the specimens were stored in distilled water at 37ºC.

Each specimen was individually fixed to a custom-made testing jig with cyanoacrylate glue (Universal Instant Adhesive; Henkel Adhesives Co. Ltd., Shantou, China). Finally, the specimens were subjected to a tensile load at a crosshead speed of 0.5 mm/min (Micro Tensile Tester; Bisco, USA) until failure occurred.

To record the failure modes, both surfaces of each fractured specimen were observed under a stereomicroscope (Nikon Eclipse E600; Tokyo, Japan) at 40x magnification. The classifications of the fracture modes were: (1) cohesive failure in the composite resin or dentin; (2) failure in the adhesive joint; (3) mixed failure. Two samples of each group were examined under a scanning electron microscope (JSM 6060F; JEOL, Tokyo, Japan).

Three-way analysis of variance (ANOVA) and t-test were used to compare the effects of the dentin bonding agents and concentrations of EGCG and storage time (24 hours vs. 6 months) on bond strength. Confidence level was set at 95% (α= 0.05). 

## Results

*FTIR*

The measurement of the degree of conversion revealed that there was no significant difference between the DC% of the adhesives with different EGCG concentrations (*p*= 0.468). The DC% of Clearfil SE Bond was significantly greater than the Filtek Silorane System (*p*= 0.001).

*Micro tensile bond strength*

The means and standard deviations of micro tensile bond strength values (MPa) of experimental and control groups are represented in Table 1. The results of three-way ANOVA test showed significant effect of type of bonding and storage duration as the main factors as well as interaction of concentration and type of bond on bond strength of the groups (*p< *0.05) (Table 2).

**Table 1:** Means and Standard Deviation of microtensile bond strength (MPa) in the group tested

**The groups according ** **to EGCG percentage**	**Experimental** **group**	**Control** **group**
Clearfil (0)	25.90 ±2.75^A^	29.58 ±4.07^a^
Clearfil (25)	24.28 ±6.59^B^	28.14 ±5.33^b^
Clearfil (50)	28.15 ±6.70^C^	29.49 ±3.29^c^
Clearfil (100)	30.32 ±3.94^D^	28.68 ±6.36^d^
Silorane (0)	22.10 ±2.74^E^	23.42 ±2.08^e^
Silorane (25)	21.03 ±2.64^F^	21.30 ±2.28^f^
Silorane (50)	17.79 ±1.63^G^	18.23±1.37^g^
Silorane (100)	17.28±2.24^H^	17.64±1.65^h^

B,D; E,G; E,H; F,G; F,H; e,f; e,g; e,h; f,g and f,h have a significant difference by Tukey’s HSD tests (*p*< 0.05).

**Table 2:** The results of three-way ANOVA of microtensile bond strength in different groups according to main factors and their interaction

**Source**	**df**	**Mean** **Squares**	**F value**	**Sig.**
Bonding	1	3241.02	212.06	.000
Storage time	1	69.49	4.54	.034
Concentration	3	36.20	2.36	.072
Bonding Vs. Storage time	1	17.53	1.14	.286
Bonding Vs. Concentration	3	167.52	10.96	.000
Storage time Vs. Concentration	3	23.69	1.55	.203
Bonding Vs. Stored time Vs. Concentration	3	17.51	1.14	.332

The means of micro tensile bond strength of groups with Clearfil SE Bond were significantly higher than those in which Filtek Silorane System was applied.

The results of t-test in Clearfil SE Bond groups in-dicated that only Clearfil (0) experimental and control groups had significant effect on bond strength (*p*= 0.021); whereas in Silorane groups, there were no significant differences between non-stored and stored groups (*p*> 0.05).

*Assessment of the failure mode*

In the Filtek Silorane System groups, adhesive failure was the most common fracture mode. In the Clearfil SE Bond groups, however, mixed and cohesive failures were the most common fracture modes (Table 3).

**Table 3:** Frequency of different fracture mode (%) in experimental and control groups

**The groups according to EGCG percentage**	**Experimental group**			**Control group**		
	**Adhesive**	**Cohesive**	**Mixed**	**Adhesive**	**Cohesive**	**Mixed**
Clearfil (0)	60	40	0	40	40	20
Clearfil (25)	40	30	30	70	20	10
Clearfil (50)	10	50	40	10	70	20
Clearfil (100)	60	40	0	30	20	50
Silorane (0)	90	10	0	20	80	0
Silorane (25)	40	60	0	40	60	0
Silorane (50)	80	20	0	90	10	0
Silorane (100)	80	20	0	90	10	0

*SEM observations*

**Figure 1:**
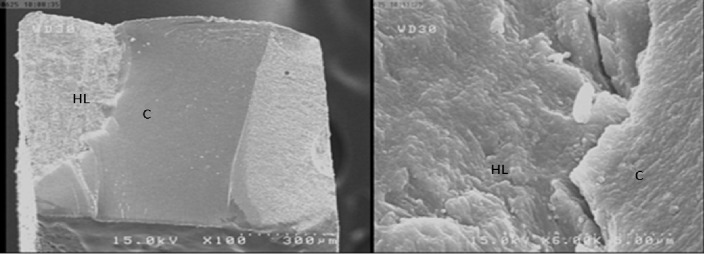
SEM views of the fractured surface in group NP2 showing mixed failure, composite resin is visible on surface (HL: Hybrid Layer C:composite) (A) ×100; (B) ×6000

**Figure 2:**
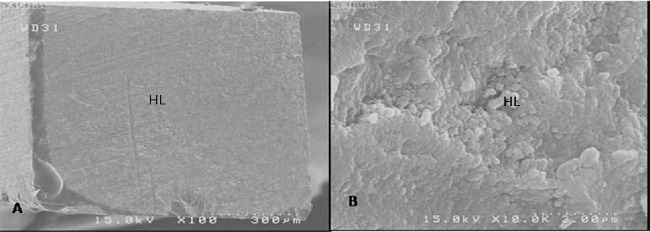
SEM views of the fractured surface in group SP1, showing adhesive failure and a uniform hybrid layer on surface. (HL: Hybrid Layer), (A) ×100; (B) ×10000

**Figure 3:**
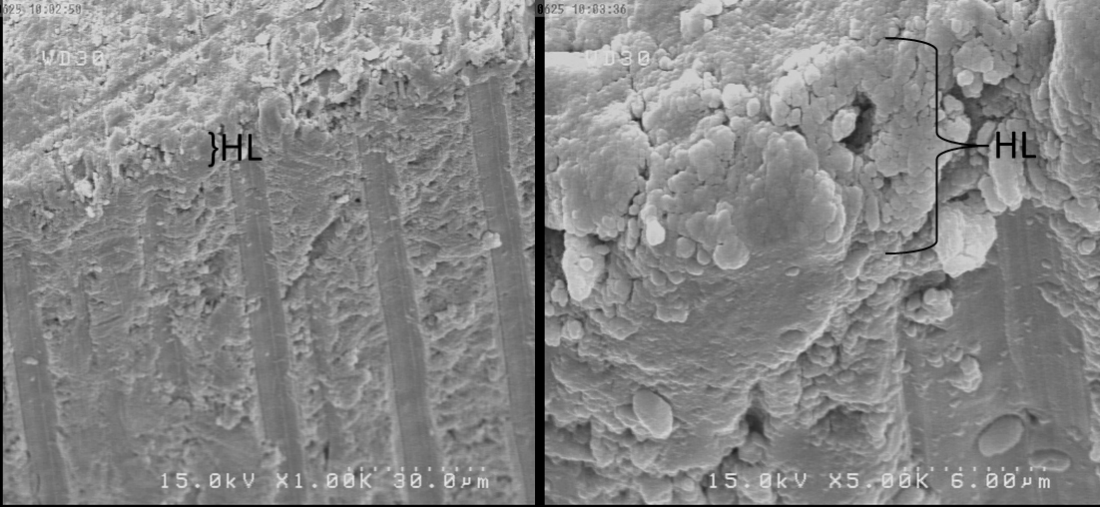
SEM views showing failure at hybrid layer or above it in Clearfil SE Bond. (HL: Hybrid Layer), (A) ×1000; (B) ×5000

The results of SEM at different magnifications showed mixed fracture mode to be the most common failure mode (figures 1 and 2). In fact, most fractures occurred in the hybrid layer (Figure 3).

## Discussion

The preservation of dentin-adhesive bonds increases the clinical efficiency of bonded restorations. In the process of bonding, however, the simplified etch-and-rinse and self-etch adhesives, reactivate the endogenous collagenolytic and gelatinolytic activities. These can be broken down into unprotected collagen fibrils in presence of water derived from water sorption by hydrophilic adhesives.[3] In this study, we used two types of two-step self-etch adhesives. Clearfil SE Bond was chosen because it had functioned well during *in vitro* and clinical studies,[15-16] and Filtek Silorane System was selected due to its novelty. Green tea polyphenols, especially epigallocatechin gallate (EGCG), have been reported to have the ability to stop the activation of proMMP-2, MMP-2 and MMP-9.[4, 11-12]

Yun *et al.* demonstrated that EGCG inhibited the activity of MMPs at 20 µM concentrations.[17] As adding different concentrations of EGCG into bonding could have various inhibitory effects on MMPs, its effect on bonding strength was also measured in 50 and 100 µM concentrations. In accordance to Du *et al.*,[14] the results of our study showed that adding EGCG into adhesives had no effect on DC.

The results of this study showed that the bond strength of Clearfil SE Bond was considerably higher than that of the Filtek Silorane System. An explanation for this could be that Clearfil SE Bond contains 10-MDP monomer, and the intense chemical adhesion to tooth tissue may be, to some extent, attributed to that.[15] Yoshida *et al.*[18] concluded that this etching which is a completely hydrophobic and a relatively hydrolysis stable monomer, is able to form strong ionic bonds with calcium because of low dissolution rate of its calcium salt in water. Additionally, these results prove that there are no considerable differences in μTBS between the groups with different concentrations of EGCG and the control group.

Furthermore, other main factors such as time of storage, significantly decreased μTBS. Studies have shown that dentin-adhesive interface stability cannot be preserved forever[19] because degradation throughout the dentin-bonded interface occurs quickly.[20-21] The mentioned reasons contain both physical and chemical factors. Physical factors include occlusal chewing forces and repetitive expansion and contraction stresses[22] associated with temperature changes within the oral cavity.[19] On the other hand, there are acidic components in dentinal fluid, saliva, food, beverages, and bacterial products, that act as chemical factors and degrade unprotected collagen fibrils[23-25] and the resin component.[20, 23-24,26-27]

The incorporation of EGCG in high concentrations decreased μTBS in the Filtek Silorane System groups. It seems that EGCG is not compatible with the formulation of Filtek Silorane System; although incorporating 25 µM EGCG with the Filtek Silorane System resulted in preservation of μTBS after 6 months. Whereas, in Clearfil SE bond groups, amounts higher than 25 µM EGCG were required to preserve μTBS after 6 months.

Zhou *et al.* in their *in vitro* study showed when chlorhexidine was incorporated in the primer of Clearfil SE bond in concentrations of 0.1٪ and higher, the dentin bond could be preserved.[28] Zhang and Kern revealed in their study that it would be advantageous to inhibit the breakdown of unprotected collagen fibrils by host-derived MMPs in the dentinal hybrid layer. Tissue inhibitors of metalloproteinases (TIMs) are the major endogenous inhibitors of MMPs when there is a balance between MMPs, TIMs, and tissue ECM (Extra Cellular Matrix).[4] Moreover, it has been shown that the acidic potential of currently-used adhesives is accountable for the breakage of unprotected collagen fibrils without bacteria. As a result, protease inhibitors that are added to primers can be recommended to enhance the stability of the dentinal collagen fibrils within the hybrid layer.[7]

In the current study, stereomicroscope observations showed that the sum of cohesive and mixed failure mode in Clearfil SE Bond groups was 74%. In addition, in Filtek Silorane System it was shown that 69% of fracture patterns were adhesive failures. As shown in this study, mixed fracture increased in the EGCG incorporated groups after six-months of storage. In a meta-analytical study,[29] a close relationship between the failure mode and the mean bond strength was revealed. Contrarily, when the bond strength is higher, a higher rate of cohesive failure is observed. The opposite is true when the bond at the dentin interface is weak; therefore, failure occurs at this location.

SEM assessment of the fractured surfaces showed that in self-etch adhesives, fracture occurred at the hybrid layer; while in the etch-and-rinse adhesives, fractures were under the hybrid layer and uninfiltrated demineralized collagen.

It seems that 50 and100 µM EGCG could preserve bond strength of the Clearfil SE Bond groups after 6 months; while EGCG especially in high concentrations could not preserve the bond strength of Filtek Silorane System samples after 6 months. The incorporation of EGCG in different amounts did not affect the degree of conversion in comparison with the control group in either adhesive system. 

Further *in vitro* and *in vivo* long-term studies are required to test the effect of EGCG on the longevity of resin-dentine interfaces. This can be done through the use of different types of self-etch adhesives. In addition, other types of MMPs can be used and compared with EGCG. Moreover, TEM observations of the hybrid layer can be suitable to directly demonstrate EGCG effects on the durability of dentin-adhesive interface.

## Conclusion

Although adding 100 µM volume of EGCG to Clearfil SE Bond can preserve the dentin bond, incorporation of EGCG in the silorane system, especially in high concentrations, decreases the bond strength after 6 months. The results of this study suggest that MMPs manufacturers can use adhesives in order to increase bond durability. 
